# Acute Toxicity of Intravenously Administered Titanium Dioxide Nanoparticles in Mice

**DOI:** 10.1371/journal.pone.0070618

**Published:** 2013-08-08

**Authors:** Jiaying Xu, Hongbo Shi, Magaye Ruth, Hongsheng Yu, Lissy Lazar, Baobo Zou, Cui Yang, Aiguo Wu, Jinshun Zhao

**Affiliations:** 1 The Faculty of Life Science of College of Science & Technology, Ningbo University, Ningbo, China; 2 Public Health Department, Ningbo University, Ningbo, China; 3 Affiliated Hospital of Medical School, Ningbo University, Ningbo, China; 4 Key Laboratory of Magnetic Materials and Devices, Division of Functional Materials and Nanodevices, Ningbo Institute of Materials Technology and Engineering (NIMTE), Chinese Academy of Sciences, Ningbo, China; IIT Research Institute, United States of America

## Abstract

**Background:**

With a wide range of applications, titanium dioxide (TiO_2_) nanoparticles (NPs) are manufactured worldwide in large quantities. Recently, in the field of nanomedicine, intravenous injection of TiO_2_ nanoparticulate carriers directly into the bloodstream has raised public concerns on their toxicity to humans.

**Methods:**

In this study, mice were injected intravenously with a single dose of TiO_2_ NPs at varying dose levels (0, 140, 300, 645, or 1387 mg/kg). Animal mortality, blood biochemistry, hematology, genotoxicity and histopathology were investigated 14 days after treatment.

**Results:**

Death of mice in the highest dose (1387 mg/kg) group was observed at day two after TiO_2_ NPs injection. At day 7, acute toxicity symptoms, such as decreased physical activity and decreased intake of food and water, were observed in the highest dose group. Hematological analysis and the micronucleus test showed no significant acute hematological or genetic toxicity except an increase in the white blood cell (WBC) count among mice 645 mg/kg dose group. However, the spleen of the mice showed significantly higher tissue weight/body weight (BW) coefficients, and lower liver and kidney coefficients in the TiO_2_ NPs treated mice compared to control. The biochemical parameters and histological tissue sections indicated that TiO_2_ NPs treatment could induce different degrees of damage in the brain, lung, spleen, liver and kidneys. However, no pathological effects were observed in the heart in TiO_2_ NPs treated mice.

**Conclusions:**

Intravenous injection of TiO_2_ NPs at high doses in mice could cause acute toxicity effects in the brain, lung, spleen, liver, and kidney. No significant hematological or genetic toxicity was observed.

## Introduction

Due to smaller size, larger surface area per unit mass and stronger catalytic activity, TiO_2_ NPs, have been widely used in many applications such as drug delivery, antibacterial materials, cosmetics and electronics [1], [2]. However, concerns have been raised that these same properties of TiO_2_ NPs may present unique bioactivity and challenges to human health [3], [4]. A rapid growth in the number of published studies confirms that there is a high level of interest concerning the safety of TiO_2_ NPs from the scientific community. Some studies have revealed that TiO_2_ NPs are more toxic than TiO_2_ fine particles [4], [5].

Recently, many investigations focused on the *in vivo* distribution of TiO_2_ NPs. Inhalation, intratracheal instillation, dermal penetration, and oral gavage are the methods most frequently used in distribution studies of TiO_2_ NPs [6]-[8]. It is worth noting that in nanomedicine, intravenous injection of TiO_2_ nanoparticulate carriers directly into the blood without passing through the normal absorption process has raised public concerns regarding their toxicity to humans [9], [10]. Ferin *et al*. [11] studied the pulmonary retention of TiO_2_ NPs and fine particles in rats after a single intratracheal instillation and a 12 week inhalation of different sizes of TiO_2_ particles (12, 21, 230, and 250 nm). Both acute and sub-chronic inhalation studies showed that TiO_2_ NPs at equivalent masses access the pulmonary interstitium to a larger extend than fine particles. They also found that the translocation process appeared to be related to the particle size, the delivered dose, and the delivered dose rate. Wang *et al*. [7] investigated the distribution of three different sizes of TiO_2_ NPs (25, 80, and 155 nm) in mice by a single oral administration. Distribution examination showed that TiO_2_ NPs were mainly retained in the liver, spleen, kidneys, and lung tissues after uptake by the gastrointestinal tract. Fabian *et al.* [12] investigated the tissue distribution after intravenous administration of TiO_2_ NPs. The levels were highest in the liver, followed by the spleen, lung, and in decreasing order, respectively. However, most of these studies did not elaborate on the toxicity of TiO_2_ NPs. In this study, mice were treated with a single intravenous injection of TiO_2_ NPs in saline suspension. Animal mortality, genotoxicity, biochemical, hematological, and histopathological effects were analyzed 14 days after treatment.

## Materials and Methods

### 1. Materials

TiO_2_ NPs were obtained from Hangzhou Wanjing new material Co, Ltd (Lot No. 20110228). The detailed particle characteristics as given by the manufacturer are shown in Table 1.

**Table 1:** Characteristics of TiO_2_ NPs used in this study.

Name	Crystal form	Surface feature	Purity (%)	Particle size (nm)	Impurities
TiO_2_ NPs	100% Anatase	Hydrophilia	99.99	40±5	Pb <2 ppm
					Cd <1 ppm
					As <1 ppm
					Hg <1 ppm
					Ni <1 ppm

Isoflurane was obtained from Sun Chemical Technology (Shanghai). Hitachi 7600–110 autoanalyzer (Hitachi, Tokyo, Japan) and blood analyzer (Sysmex XT-1800i, Japan) were used for biochemical and hematological analysis, respectively. ICR mice were purchased from Zhejiang Province Laboratory Animal Science Center.

### 2. TiO_2_ NPs preparation

A stock suspension of TiO_2_ NPs was prepared in saline (10 mg/ml) by sonication for 30 seconds using a Branson sonifier 450 (Branson Ultrasonics Corp., Danbury, CT). The particle suspensions were kept on ice for 15 seconds and sonicated again on ice for a total of 3 minutes at a power of 400 W. Before use, TiO_2_ NPs were diluted to desired concentrations in fresh saline. All samples were prepared under sterile conditions.

### 3. Detection of the size distribution

Size distribution of TiO_2_ NPs was captured using scanning electron microscopy (SEM) (Hitachi S-4800; Japan). Briefly, TiO_2_ NPs were prepared by sonication. The samples were then diluted in double-distilled water and air-dried onto a carbon planchet. Images were collected on a SEM. Optimas 6.5 image analysis software (Media Cybernetics; Bethesda, MD) was used to measure the diameter of TiO_2_ NPs.

### 4. Animal husbandry and treatment

48 (24 male and 24 female) ICR mice were housed according to their sex in stainless steel cages in a ventilated animal room (Relative humidity at 60±10% and a 12 hour light/dark cycle). Room temperature was maintained at 20±2°C. Distilled water and sterilized food for mice were available *ad libitum*. Animal study protocols were approved by the Ningbo University Institutional Animal Care and Use Committee.

Animals were randomly divided into 5 groups: 1 control group and 4 experimental groups (0, 140, 300, 645, and 1387 mg/kg BW of TiO_2_ NPs). Each group had 8 mice (4 male and 4 female). The remaining 8 mice (4 male and 4 female) were used as the positive control in the micronucleus test. TiO_2_ NPs dose setting was based on the principle of Horn's Method. After mixing with a vortex, a single injection of TiO_2_ NPs saline suspension was administered through the tail vein (28 G needle). Control group mice were given saline only. Behavior and mortality were monitored and recorded carefully after treatment.

### 5. Anesthesia and necropsy

14 days after treatment, blood samples were collected from the femoral artery by a quick incision of the artery while the animal was anesthetized with 2% isoflurane. The animals were then euthanized by cervical dislocation. Serum was obtained by centrifugation at 3500 rpm for 10 minutes and stored at −80° until used. Bone marrow smears were prepared by smearing a mixture of mouse bone marrow from the femur and calf serum on a coated glass slide. Slides were air dried and then fixed in methanol for 15 minutes for the micronucleus test. The organs (heart, lung, liver, spleen, kidneys and brain) were excised and weighed accurately. A small piece of tissue from each organ was dissected, fixed in a 6% formalin solution and stored at 4°C until used.

### 6. Organ weight/BW coefficients

After weighing the organs, the organ weight/BW coefficients of heart, lung, liver, spleen, kidneys and brain were calculated as organ weight (wet weight, mg)/BW (g)×100%.

### 7. Blood biochemical analysis

Biochemical parameters detected in blood serum of mice included total bilirubin levels (TBIL), indirect bilirubin (IBIL), direct bilirubin (DBIL), alkaline phosphatase (ALP), alanine aminotransferase (ALT) and aspartate aminotransferase (AST), blood urea nitrogen (BUN), creatinine (CREA), uric acid (URCA) and the enzyme creatine kinase (CK).

### 8. Hematology analysis

Hematological parameters examined in this study included WBC count, red blood cell (RBC) count, hemoglobin (HGB), red blood cell specific volume (HCT), mean corpuscular volume (MCV), mean corpuscular hemoglobin (MCH), mean corpuscular hemoglobin concentration (MCHC), red cell distribution width (RDW-CV), platelet (PLT), platelet distribution width (PDW-CV), mean platelet volume (MPV) and plateletcrit (PCT).

### 9. Histopathology examination

The fixed tissues were stored at 4°C overnight and then embedded in paraffin blocks. 4 μm thick tissue sections were prepared and stained with hematoxylin and eosin (H&E). Histopathological morphology was checked under the microscope by an independent pathologist.

### 10. Micronucleus test

The methanol fixed bone marrow smears were stained with Giemsa stain according to the conventional method. One thousand polychromatic erythrocytes per animal were analyzed for the presence of micronucleus in cells. Cyclophosphamide (20 mg/kg, two intraperitoneal injections, at 24 and 48 h before mice were sacrificed, respectively) was used as a positive control.

### 11. Statistical analysis

Results were expressed as mean ± standard deviation (SD). Multigroup comparisons of the means were carried out by one-way analysis of variance (ANOVA) test. Dunnett's test was used to compare the difference between the experimental groups and the control group. The statistical significance for all tests was set at *P*<0.05.

## Results

### 1. The average size distribution of TiO_2_ NPs

**Figure 1:**
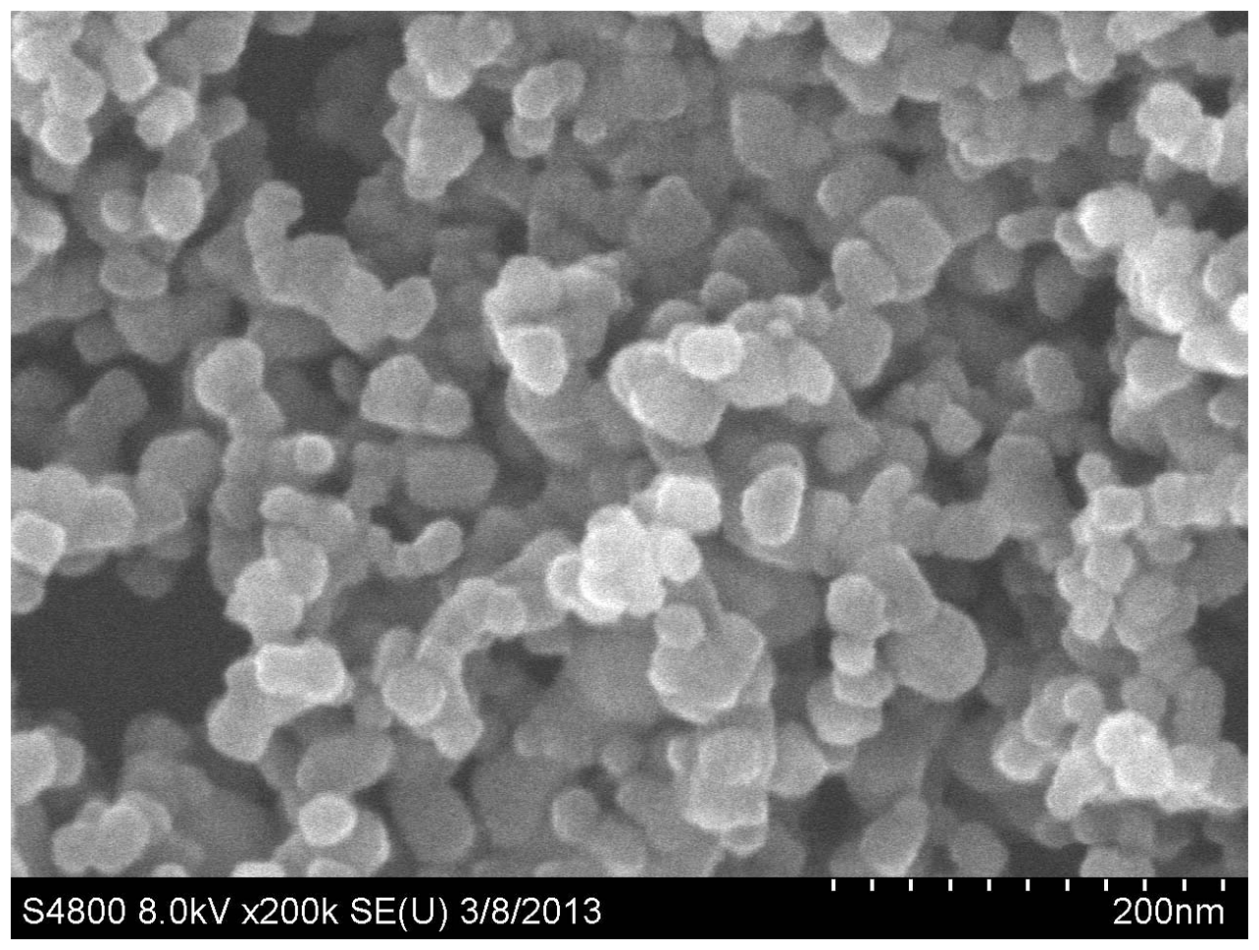
The image of TiO_2_ NPs was captured by SEM.

**Figure 2:**
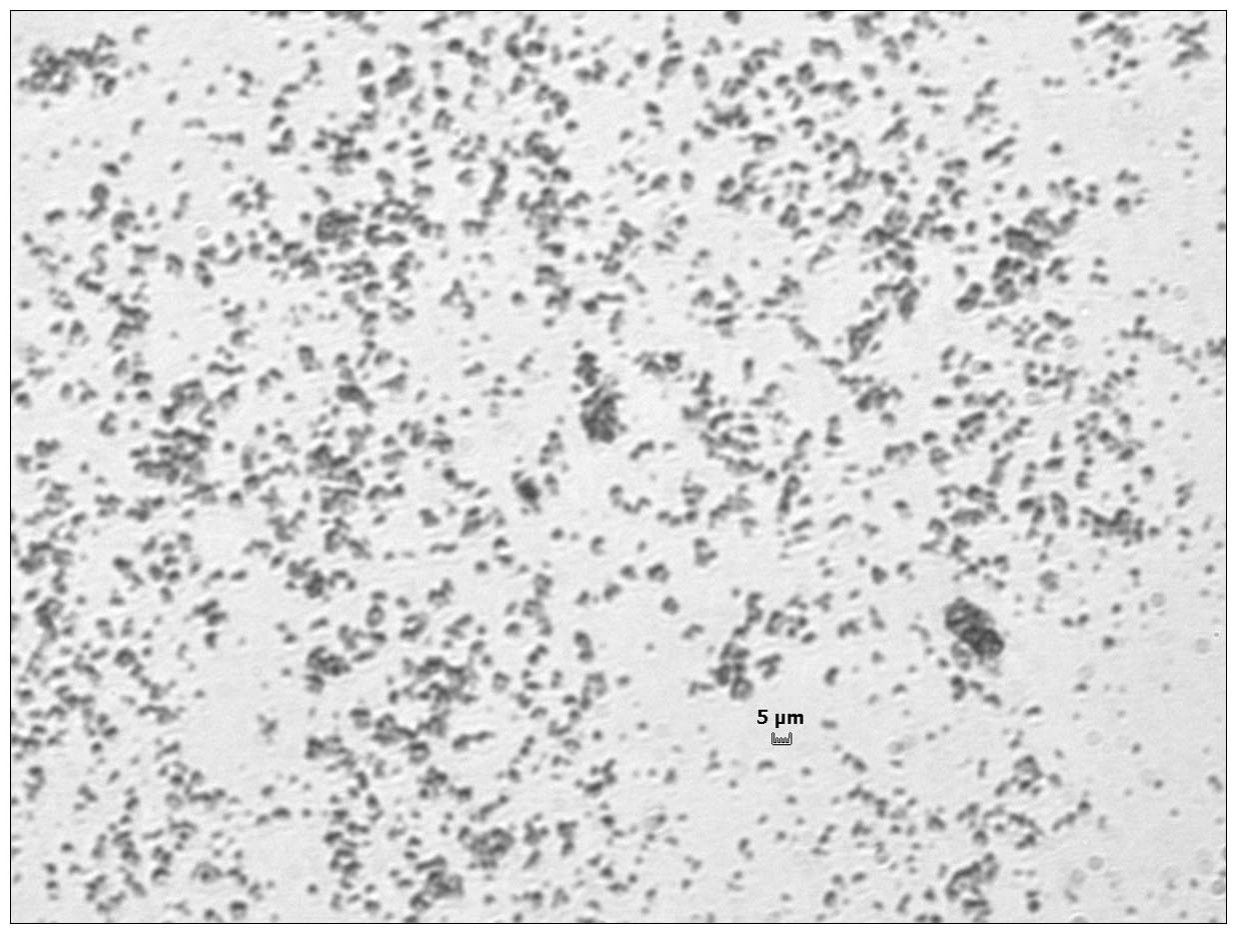
The image of TiO_2_ NPs aggregates in saline under the light microscope. TiO_2_ NPs in saline solution was dropped onto a glass slide and the aggregates of TiO_2_ NPs in solution was checked under the microscope (400×). After precipitation, the larger aggregates were in the size range of 100–500 nm, and the smaller aggregates were not visible under the light microscope at 400×.

The average size distribution of TiO_2_ NPs was 42.30±4.60 nm as detected by optimas 6.5 image analysis software. Image of TiO_2_ NPs was captured by SEM (Figure 1). The size distribution of TiO_2_ NP aggregates in saline was observed under the light microscope (Figure 2).

### 2. Behavior, symptoms and mortality

7 days after treatment, difference in eating and drinking patterns and physical activity were observed in the 1387 mg/kg dose group. They showed decreased food and water intake and decreased physical activity than control group. At day 9, 2 males and 4 females in the 1387 mg/kg dose group died. 2 males survived at this dose level, leaving less than three mice at the end of the experiment. Therefore, in this dose group only the tissues were histopathologically analyzed.

### 3. Organ weight/BW coefficients

Data were expressed as means ± SD (n = 4) (Table 2). Comparisons were carried out by one-way variance test (SNK's multiple comparison tests). Compared to control, coefficients of the spleens in TiO_2_ NPs treated mice increased significantly; however, coefficients of the liver and kidney decreased significantly. No significant effects were observed in the coefficients of the heart, lung or brain in TiO_2_ NPs treated mice.

**Table 2:** Organ weight/BW coefficients (Mean ± SD).

Indexes	TiO_2_ NPs (mg/kg)			
	0	140	300	645
Heart/BW (mg/g)	4.97±0.90	4.96±0.60	5.41±0.33	5.16±0.60
Lung/BW (mg/g)	6.52±0.93	6.30±0.29	7.61±1.25	7.19±1.51
Liver/BW (mg/g)	54.05±3.91	55.93±5.09	47.25±5.68*	46.21±4.70*
Spleen/BW (mg/g)	4.03±0.10	13.05±2.72*	9.26±4.59*	7.41±5.07*
Kidney/BW (mg/g)	14.66±2.36	4.75±0.95*	9.21±5.28*	13.05±5.69
Brain/BW (mg/g)	14.51±1.88	13.41±1.50	14.69±1.89	12.24±2.73

* Significantly different compared to control.

### 4. Blood biochemical analysis

Biochemical parameters in the serum as detected by the autoanalyzer are listed in Table 3. No significant differences were found in the serum levels of TBIL, ALT, AST, ALP, BUN, CREA, or CK in TiO_2_ NPs treated mice as compared to the control group. The levels of DBIL and IBIL in TiO_2_ NPs treated mice decreased in a dose dependent manner. The level of URCA in TiO_2_ NPs treated mice at 140 and 300 mg/kg were significantly increased compared to the control group.

**Table 3:** Biochemical parameters in blood serum (Mean ± SD).

Indexes	Control	TiO_2_ NPs (mg/kg)		
		140	300	645
TBIL	2.98±0.05	1.54±0.61	2.33±0.602	1.43±0.56
DBIL	1.73±0.26	1.58±0.36	1.08±0.19**	0.80±0.42**
IBIL	1.93±0.15	1.425±0.95	1.25±0.65*	0.63±0.36*
ALT	38.00±3.27	42.75±12.74	52.00±25.21	41.33±10.50
AST	135.25±35.51	180.33±133.16	165.88±65.77	118.00±79.25
ALP	60.50±31.6	54.25±12.82	47.38±20.52	57.00±32.09
BUN	10.74±3.34	7.28±0.544	8.16±0.71	7.42±0.59
CREA	6.00±0.00	4.63±2.46	6.38±1.49	7.00±2.94
URCA	129.00±34.32	219.25±53.27*	175.25±15.78*	155.00±29.24
CK	5006.25± 1576.28	5743.00± 1766.35	5907.00± 5366.74	6442.00± 4412.18

* Significantly different compared to control.

** Significantly different compared to control and 140 mg/kg group.

### 5. Hematological analysis

Hematological parameters in the blood as detected by the autoanalyzer are listed in Table 4. Intravenous injection of high doses of TiO_2_ NPs did not induce significant acute hematological toxicity except for an increase in the WBC count in 645 mg/kg dose group.

**Table 4:** Hematological parameters in the blood (Mean ± SD).

Indexes	Control	TiO_2_ NPs (mg/kg)		
		140	300	645
WBC	5.00±0.93	7.00±2.21	7.20±2.52	7.58±2.57*
RBC	9.30±0.39	9.53±0.74	9.87±0.48	8.88±0.17
HGB	142.25±5.56	145.33±7.37	153.00±7.07	141.00±4.58
HCT	0.48±0.024	0.37±0.22	0.49±0.02	0.48±0.02
MCV	51.75±0.58	51.98±3.97	48.95±0.64	54.43±2.61
MCH	15.33±0.39	12.83±4.97	15.50±0.00	15.87±0.72
MCHC	295.50±5.92	304.00±6.25	316.50±3.54	291.667±2.08
RDW-CV	20.35±0.56	19.20±3.33	20.85±0.35	19.80±0.87
PLT	1072.25± 128.67	1083.00± 292.12	1074.50± 85.56	1155.33± 190.54
PDW-CV	7.18±0.25	7.00±0.42	7.25±0.07	7.03±0.06
MPV	6.45±0.19	6.63±0.26	6.50±0.00	6.60±0.27
PCT	0.69±0.10	0.54±0.37	0.70±0.06	0.77±0.15

* Significantly different compared to control.

### 6. Histopathological detection

**Figure 3:**
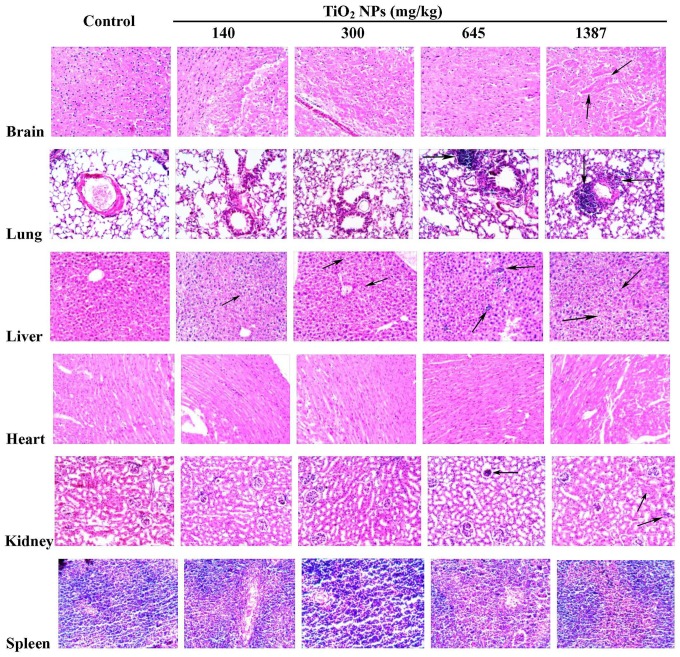
Pathological morphology of different tissues observed under microscope. In mice treated with TiO_2_ NPs, neuronal cell degeneration was observed in the brain tissue; vacuoles were observed in the neurons of hippocampus and their number was increased in the high dose groups, which indicated fatty degeneration occurred in the hippocampus of brain tissue. In the lung tissues, perivascular infiltration of inflammatory cells, foamy cells as well as pulmonary fibrosis were observed; the granulomatous lesions were found at the doses of 645 and 1387 mg/kg. At the doses of 140 and 300 mg/kg, TiO_2_ NPs showed vacuolar degeneration in the liver. At 645 mg/kg, inflammatory cells were found in the bile ducts of the liver, and hydropic degeneration around the central vein and spotty necrosis of hepatocytes were also observed. At 645 and 1387 mg/kg, multifocal lesions were observed in the liver. In the kidneys, swelling in the renal glomerulus was observed in TiO_2_ NPs treated mice. In the spleen, minor lesions were observed due to increased proliferation of local macrophages. No obvious abnormality in histology was observed in the heart in TiO_2_ NPs treated mice as seen under the microscope (400×).

Histopathological examination of the tissues indicated that intravenous administration of high doses of TiO_2_ NPs could induce multi-organ pathological lesions in a dose dependent manner (Figure 3). The results show that TiO_2_ NPs treatment could induce different degrees of damage in the brain, lung, spleen, liver and kidneys. However, no obvious pathological effects were observed in the heart of TiO_2_ NPs treated mice.

### 7. Micronucleus test

**Figure 4:**
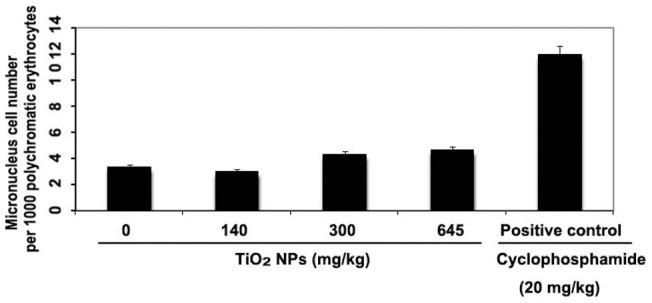
Micronucleus test result. Average of micronucleus cell number per thousand polychromatic erythrocytes in animals was analyzed. No significant difference was observed among the micronucleus cell number between TiO_2_ NPs treated mice and the control.

Micronucleus test result 14 days after a single intravenous injection of different doses of TiO_2_ NPs shows, no significant increase in micronucleus cell number in the polychromatic erythrocytes among TiO_2_ NPs treated mice as compared to the control (Figure 4).

## Discussion

Studies have demonstrated that accumulation of TiO_2_ NP can be observed in the liver, lung, kidneys, and spleen after intraperitoneal, intravenous or dermal administration.[13]–[15] After inhalation exposure in rats, TiO_2_ NPs have been found to accumulate in the lung, leading to phagocytosis [16], [17]. Wang *et al.* [18] found that a single oral gavage exposure of a very high dose (5 g/kg) of TiO_2_ NPs (80 nm) in mice could elevate the ALT/AST enzyme ratio and LDH level in serum, which implies TiO_2_ NPs may induce hepatic injury. Fabian *et al.* [12] reported the accumulation of TiO_2_ NPs in the liver, spleen, lung, and kidneys after intravenous administration of 5 mg/kg TiO_2_ NPs in rat. However, there were no remarkable toxic effects observed in these organs.

In this study, behavioral and physical symptoms of acute toxicity such as decreased activity or decreased uptake of food and water were observed in the first week in the mice treated with 1387 mg/kg of TiO_2_ NPs. The behavior of the mice in the other dose groups was normal throughout the study. Compared to the control group, organ coefficients of the spleens in TiO_2_ NPs treated mice increased significantly; while, the organ coefficients of the liver and kidneys decreased significantly. Both increase and decrease of the organ coefficients may be caused by TiO_2_ NPs excretion or accumulation in the organs, which could cause certain histopathological changes. These changes were confirmed in the histopathological tissue sections of the liver and kidneys. The liver tissue showed hepatocyte vacuolar degeneration, spotty necrosis of liver hepatocytes, along with inflammatory cell invasions in the bile ducts of the liver, and hydropic degeneration around the central vein. At higher doses, multifocal lesions were also observed in the liver. Wang *et al.* [18] reported that TiO_2_ NPs treatment through oral administration could increase hepatocyte necrosis. Combined with our results, it can be concluded that TiO_2_ NPs can induce hepatocyte injury *in vivo*.

In the kidneys, swelling in the renal glomerulus in TiO_2_ NPs treated mice was observed. Serum biochemical analysis showed a significant increase in blood URCA level in TiO_2_ NPs treated mice. URCA is the end product of decomposition of a purine nucleic acid, which is mainly excreted from the kidneys. Blood URCA levels are elevated in renal dysfunction [19]. In the spleen, TiO_2_ NPs treatment caused increased proliferation of local macrophages, which is consistent with the results of the increased organ coefficients.

Although no significant changes were found in the organ coefficients for brain and lung tissues in TiO_2_ NPs treated mice, histopathological examination showed certain lesions. The brain tissue showed neuronal cell degeneration and vacuoles were observed in the hippocampus, which is indicative of fatty degeneration in the hippocampus. In the lung tissues, inflammatory cells, foamy cells and granulomatous lesions were observed. Park *at al.* [20] reported that granulomatous lesions were found in the bronchiole and alveoli of the lung after 14 days TiO_2_ NPs treatment (5 mg/kg, 20 mg/kg, and 50 mg/kg) in mice. Roursgaard *et al.* [21] also found inflammation in the lungs after TiO_2_ NPs treatment in mice by intratracheal instillation.

Bilirubin consists of TBIL, IBIL, and DBIL. Studies have revealed that the levels of serum bilirubin are inversely related to the risk of certain heart diseases [22]. The results showed that both IBIL and DBIL significantly decreased in TiO_2_ NPs treated mice compared to the control mice, which suggests that TiO_2_ NPs treatment might also have myocardial effects. However, histopathological examination of the heart showed no pathological effects and no significant changes in the organ coefficients in the TiO_2_ NPs treated mice in this study. These differences may be attributed to late onset of histological evidence of myocardial pathology.

Hematological analysis is normally used to detect the hematological toxicity of different chemicals. In this study, the results indicated that, 14 days after intravenous administration of the high dose of TiO_2_ NPs, no significant hematological toxicity could be observed.

The micronucleus test is frequently used as a tool for genotoxicity assessment of various chemicals. It is easier to conduct than the chromosomal aberration test in terms of procedures and evaluation. In this study, 14 days after a single intravenous injection of different doses of TiO_2_ NPs, there was no significant increase in the number of micronucleated cells in the TiO_2_ NPs treated mice compared to the control.

In summary, after a single intravenous injection of different doses of TiO_2_ NPs, animal mortality, blood biochemistry, hematology, genotoxicity and histopathology were investigated 14 days after treatment. Mice died on day 2 of the study and acute toxicity symptoms such as decreased physical activity or decreased food and water intake were observed in the first week in the high dose group (1387 mg/kg). Hematological analysis and micronucleus test showed no significant acute hematological or genetic toxicity except an increase of WBC count in mice at 645 mg/kg. The mice treated with TiO_2_ NPs showed significantly higher spleen organ weight/BW coefficients, and lower liver and kidney organ/BW coefficients compared to control. Serum biochemical parameters and histological detections indicated that TiO_2_ NPs treatment could induce different degrees of damage in the brain, lung, spleen, liver and kidneys. No pathological changes were observed in the heart in the TiO_2_ NPs treated mice. Further evaluation for chronic toxicities after intravenous injection of TiO_2_ NPs in animals is still needed.
